# Reward type and behavioural patterns predict dogs’ success in a delay of gratification paradigm

**DOI:** 10.1038/srep42459

**Published:** 2017-03-08

**Authors:** Désirée Brucks, Matteo Soliani, Friederike Range, Sarah Marshall-Pescini

**Affiliations:** 1 Comparative Cognition, Messerli Research Institute, University of Veterinary Medicine Vienna, Medical University of Vienna, University of Vienna, 1210 Vienna, Austria

## Abstract

Inhibiting an immediate behaviour in favour of an alternative but more advantageous behaviour has been linked to individual success in life, especially in humans. Dogs, which have been living in the human environment for thousands of years, are exposed to daily situations that require inhibition different in context from other non-domesticated species. One task regularly used to study inhibitory control is the delay of gratification task, which requires individuals to choose between an immediate option of lower value and a delayed option of higher value. We tested sixteen dogs in a non-social delay of gratification task, conducting two different conditions: a quality and a quantity condition. While the majority of dogs failed to wait for more than 10 s, some dogs tolerated delays of up to 140 s, while one dog waited for 15 minutes. Moreover, dogs had more difficulties to wait if the reward increased in terms of quantity than quality. Interestingly, dogs were able to anticipate the delay duration and some dogs developed behavioural patterns that predicted waiting, which seems similar in some respects to ‘coping-strategies’ found in children, chimpanzees and parrots. Our results indicate that strategies to cope with impulsivity seem to be consistent and present across animal taxa.

The ability to inhibit an immediate action in favour of a more advantageous alternative behaviour has been termed ‘inhibitory control’ in the literature. For example, it is advantageous for a stalking predator to inhibit the impulsive action of chasing every animal that it sees and instead wait, select the prey, take into account multiple parameters and only then make a decision. Still, inhibitory control is a rather complex construct^1^,^2^ with different domains that involve various aspects of behaviour. One aspect of inhibitory control is self-control or behavioural inhibition, i.e. controlling an impulsive behaviour in a tempting situation^2^.

In humans, self-control is thought to be an important predictor for many aspects of life. Persons with better self-control are more successful in life^3^, show more cooperative behaviours^4^ and are better at problem solving (e.g. ref. 5). While research on inhibitory control has its roots in human psychology, a number of studies have also been carried out to understand the role of inhibitory control in non-human animals. Dogs represent a particularly interesting species to study in this context, because they have been selected for thousands of years^6^ to live in a human environment and are therefore exceptionally good in interacting with humans and handling different situations that non-domesticated animals would have great difficulty with (e.g. walking through a park with children playing and eating all around them). In addition, dogs not only excel over many other species in understanding human social cues (for a review see ref. 7), but may also inhibit their own preferences and make counterproductive choices in favour of their owners^8^,^9^. Indeed it has been proposed that during the process of domestication dogs were selected against fear and aggression and consequently dogs possess a less reactive and tamer temperament (‘Emotional reactivity hypothesis^10^’), allowing them to inhibit immediate behaviours in favour of more delayed rewards (‘Synergistic hypothesis^11^’). Based on the above, we would therefore expect dogs to show enhanced inhibitory control abilities compared to other species. However, considering the close cohabitation with humans, the influence of training/experience might mask/confound dogs’ natural self-control abilities. A dog can be trained to inhibit eating food from the ground (using reinforcement and punishment contingencies); however, this does not imply that the dog is actively showing self-control. Rather it may be expressing its willingness to obey the human’s commands (a form of social inhibition) and/or simply show the animal’s preference to avoid reprimand (i.e. social conflict with the human) over obtaining a food morsel. Consequently, to assess dogs’ natural capacity for self-control it is important to control for, as much as possible, the potential effect of prior learning contexts and the social influence of the human partner.

Self-control is most often assessed as the ability to delay gratification – the capacity to reject an immediate reward in favour of a delayed but better reward. The delay of gratification paradigm has been widely used to evaluate self-control abilities both across species and in terms of individual variation (e.g. ref. 12). Most studies used an exchange paradigm in which individuals are required to hold the initial lower value reward for the waiting duration before they are offered the chance of exchanging it again for a reward of higher value.

Also dogs have been tested in a delay of gratification paradigm using this exchange method^13^. Results revealed that dogs tolerated surprisingly high delay times of up to 18 minutes, hence excelling most primate species (except for chimpanzees and macaques^14^,^15^), but also showing enormous individual variation in dogs’ ability to delay gratification (range from 10 to 640 seconds^13^). Nonetheless, the exchange paradigm adopted by this study required dogs to go through a lengthy training process and during the test the dogs were required to hold the low value reward in their mouth whilst the experimenter faced the dog and held the higher value reward up during the delay period. Considering dogs’ sensitivity to human cues^7^ and the lengthy training procedure involved in this paradigm, it is possible that the extraordinary delays observed were not the result of dogs’ spontaneous self-control abilities but were rather the result of social inhibition whereby the mere presence of the experimenter - which in two cases was even the owner - affected the dogs’ choice to wait (see discussion above). In order to disentangle dogs’ performance in the delay of gratification task from a human induced social inhibition, dogs would need to be tested in a different, more or less asocial setup (e.g. automated presentation of items^16^,^17^). Although one study already considered this problem and tested dogs in an asocial setup^18^, no conclusions could be drawn regarding social inhibition towards the experimenter, since this study utilized a different procedure (i.e. delay choice task), in which dogs were not required to maintain their decision to wait throughout the delay phase.

Several factors have been shown to affect performance in delay of gratification tasks (e.g. experimenter presence^16^, visibility of rewards^19^ or task context^20^) in other species. In particular, it has been consistently found that it is easier for animals to tolerate higher delays when the delayed reward is of better quality, whereas animals have more problems to wait for rewards of higher quantity (e.g. refs 21, 22, 23). However, also individual variation in performance has consistently emerged (e.g. refs 21, 22, 23) but has only rarely been investigated (but see ref. 18). In humans, different behavioural patterns adopted during the delay^24^,^25^ have been related to an individual’s capacity to wait. For example, children who found ways of distracting themselves, such as playing, were better at waiting for the more valued reward, than children who showed behaviours focused directly on the lower value reward available^25^. This aspect has received only scarce attention in non-human animals, with only two studies - one with Chimpanzees^26^ and another with a Grey parrot^27^ revealing similar distraction patterns.

In order to better assess dogs’ natural inhibitory control abilities, and evaluate whether they are indeed particularly good at waiting compared to other species we tested dogs in a non-social version of the delay of gratification task and kept the pre-test training regime at a minimum. In removing the social aspect (i.e. owner and experimenter) from the test setup and omitting social feedback, as well as presenting a context which dogs had no prior familiarity with, we aimed at minimizing the effect of human social inhibition and potentially learned inhibitory patterns associated with training/prior experience. Dogs were placed in an enclosure and rewards were delivered via bowls from behind a curtain, which hid both the experimenter and the owner during testing. Dogs had the choice of accepting the lesser-valued option at any point during the delay, since this was in reach the entire time. If they refrained from taking the low value reward for the established delay, the second bowl containing the high value reward was moved from within sight to within reach of the dog.

The second aim of the study was to evaluate what factors may affect dogs’ capacity to tolerate longer delays. Similarly to other studies (e.g. refs 21 and 23) we therefore tested dogs in two conditions: a) a quality condition, in which dogs were offered a delayed reward of better quality and b) a quantity condition, in which a higher quantity of the same reward was made accessible after a certain delay. Furthermore, considering the effect of behavioural strategies on maximum delay tolerated^24^,^26^, we carried out an in depth analyses of dog’s behaviour whilst they waited. We evaluated whether certain behaviours such as keeping a distance from the bowls, looking away, and lying down may positively affect the waiting success of dogs in the delay task.

Finally, to explore the potential relationship between dogs’ performance in the delay of gratification task to inhibitory control in every day life situations, we asked owners to compile a previously validated questionnaire (Dog Impulsivity Assessment Scale^28^) and evaluated the dogs’ performance on both these measures.

## Results

### Impulsivity Questionnaire

All dog owners rated their dogs’ impulsivity in the DIAS questionnaire (overall score: mean 0.54, range: 0.38–0.67; behavioural regulation: mean 0.50, range: 0.26–0.68; aggressiveness: mean 0.44, range: 0.20–0.68; responsiveness: mean 0.67, range: 0.45–1.00; see Supplementary Table S1). In accordance with the original study, the components aggressiveness and responsiveness were negatively correlated (Spearman: *r*_*s*_ = −0.43, *p* = 0.02). However, the other components were not correlated with each other (Spearman: behavioural regulation - aggressiveness: *r*_*s*_ = −0.22, *p* = 0.25, behavioural regulation – responsiveness: *r*_*s*_ = 0.06, *p* = 0.76).

### Maximum Delay Reached

Dogs tolerated a maximum delay of 140 seconds in the quality condition (mean ± SE = 35.6 ± 48.0 s) and 920 seconds in the quantity condition (mean ± SE = 82.9 ± 228.2 s). The dog that reached the highest delay stage in the quantity condition was not tested in the quality condition due to the owner discontinuing the test due to reasons unrelated to the study. Moreover, since his performance clearly marked an outlier to the rest of the data (i.e. maximum delay performance exceeded mean by more than three standard deviations), we decided to exclude him from all following analyses.

**Figure 1:**
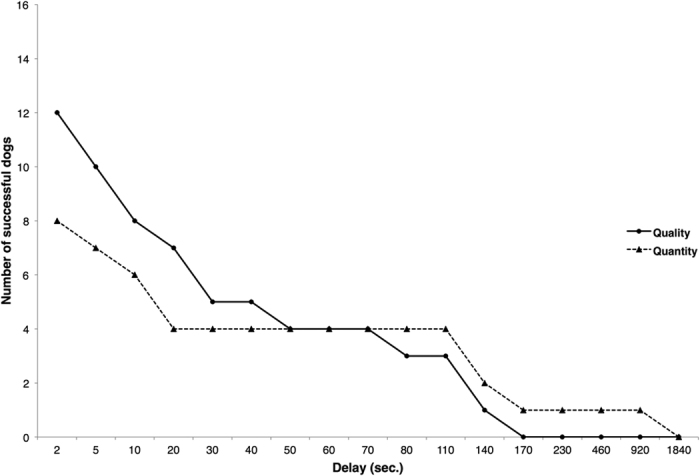
Number of successful dogs (=waiting for a minimum of 3 trials for the delayed reward option) per delay in quantity condition (N = 16) and quality condition (*N* = 15).

We found that dogs reached different maximum delays in the two test conditions (LRT: *χ*^*2*^ = 4.71, *df* = 1, *p* = 0.030; see Fig. 1). Dogs reached a lower maximum delay in the quantity condition than in the quality condition (CLMM: *β* = −1.80, SE = 0.89, *z* = −2.02, *p* = 0.044). Neither age, sex, start condition nor DIAS scores could explain dogs’ maximum waiting performance (LRT: age: *χ*^*2*^ = 2.25, *df* = 1, *p* = 0.134; sex: *χ*^*2*^ = 0.99, *df* = 1, *p* = 0.320, start condition: *χ*^*2*^ = 0.64, *df* = 1, *p* = 0.425, DIAS – overall score: *χ*^*2*^ = 2.58, *df* = 1, *p* = 0.108, DIAS – behavioural regulation: *χ*^*2*^ = 0.94, *df* = 1, *p* = 0.333, DIAS – aggressiveness: *χ*^*2*^ = 2.39, *df* = 1, *p* = 0.123, DIAS – responsiveness: *χ*^*2*^ = 2.52, *df* = 1, *p* = 0.112).

### Factors Influencing Dogs’ Waiting Performance

In the next step we investigated which factors influenced dogs’ success (i.e. number of trials waiting for delayed reward) in each test session separately from their maximum performance. We found an interaction between delay and test condition (LMM: F_12,216_ = 3.257, *p* < 0.001) and thus we analysed the two conditions separately.

In the quality condition, dogs were less successful with increasing delays (LMM: *F*_12,110_ = 18.048, *p* < 0.001, see Table 1 and Supplementary Table S2). Moreover, dogs that looked away from the food during the waiting time showed a higher success rate (LMM: *F*_1,109_ = 8.469, *p* = 0.004, see Table 1 and Supplementary Table S2). Also the proximity to the food as well as the dogs’ posture during the trial affected the waiting performance. Dogs that waited at a larger distance from the food (LMM: *F*_1,109_ = 6.959, *p* = 0.010) and dogs that waited in a laying position waited more often (LMM: *F*_1,110_ = 51.049, *p* < 0.001, see Table 1 and Supplementary Table S2). None of the three fixed effects (look away, lying and distant) was correlated with each other (all *r* < 0.32). Additionally, the number of tests conducted per test day did not affect dogs’ success rate in a session (see Table 1).

In the quantity condition dogs’ decision to wait was best explained by an interaction between the delay duration and the looking away behaviour (LMM: *F*_10,43_ = 3.251, *p* = 0.003; see Supplementary Table S3). A turning point occurs for dogs at the 50 s delay. For lower delays a negative interaction emerges with dogs that look away being less successful, however, for the higher delay stages we found a positive interaction with dogs that looked away being more successful at waiting (see Supplementary Table S3). Interestingly, we neither found an effect of proximity to bowls nor posture (see Table 1) on the success ratio in the quantity condition. Additionally, the number of tests conducted per test day did not affect dogs’ success rate in each session (see Table 1). Moreover, in the quantity condition we found that the reward type additionally affected dogs’ waiting success (LMM: *F*_1,213_ = 7.036, *p* = 0.009) and dogs were more successful in waiting for the higher quantity in LVR trials (i.e. choice between 1 piece of LVR vs. 5 pieces of LVR) than in HVR trials (i.e. choice between 1 piece of HVR vs. 5 pieces of HVR; see Supplementary Table S4). However, there was no interaction between delay stage and success in LVR and HVR trials (LMM: *F*_15,198_ = 0.886, *p* = 0.581).

### Error Times

Seven out of 10 dogs gave up waiting earlier than expected by a constant chance of giving up during a trial (=chance level) in the quality condition, whereas in the quantity condition six out of 7 dogs renounced waiting earlier than predicted by chance level (see Supplementary Table S5 for detailed results). This indicates that the majority of dogs could anticipate the delay duration and consequently decided to eat the immediate reward at the beginning of a trial instead of giving up at a random point.

## Discussion

Dogs showed a low tolerance for delays at a group-level, with half of the dogs waiting a maximum of 10 s in the quality condition and 2 s in the quantity condition. However, some dogs managed to wait for up to 140 s for a better reward in both conditions, while one exceptional dog succeeded in tolerating a delay of 15 minutes. Hence, revealing enormous individual variation in their ability to wait for a more valued reward. In addition, the majority of dogs could anticipate the delay duration, like chimpanzees^29^, corvids^30^, capuchins and macaques^14^, and made their decision to wait or to give up early during a trial. In line with findings from other studies^21^,^22^,^23^,^30^,^31^, it was easier for dogs to wait if rewards were of higher quality than larger quantity. Besides, behaviour patterns in the delay maintenance phase (i.e. distance from food, a calm posture and looking away from the reward) influenced dogs’ success in the delay task.

Compared to the dogs’ performance in the previous study^13^, the dogs in our study waited less. In the quantity condition of our study (the most comparable to ref. 13) dogs on average tolerated delays of 83 seconds, while the dogs in Leonardi *et al*.’s study waited on average three times as long (i.e. 247 seconds). This discrepancy in results suggests that the direct interaction with the experimenter in the Leonardi *et al*.’s study could have caused the dogs to view the task as a social obligation, “obeying” for a longer time compared to when the task was conducted in the absence of an experimenter, like in our study. In this respect, the current task may be more appropriate to evaluate dogs’ spontaneous ‘self-control’ abilities. However, aside from the ‘social’ aspect other factors could also be responsible for the differences in tolerated delays between the studies, for example the extensive training required for the exchange paradigm as well as the different criteria used for passing on to the next delay stage (i.e. Leonardi *et al*. waiting in one trial vs. our study waiting in three trials). Interestingly however, there is also an important similarity in results between the two studies. In line with Leonardi *et al*. we found that most of the dogs’ decision to give up waiting was made early during a trial instead of at a random point, indicating that the dogs could anticipate the delay duration of the trial and considered this as well as the value of the delayed reward when making their choices.

In general, dogs’ delay abilities shown in the current study are below most primate but also bird species that utilized a similar paradigm (e.g. Chimpanzees^29^; Long-tailed macaques^15^; Tonkean macaques^14^, Rhesus monkeys^32^, Cockatoos^21^, Grey Parrot^27^). At the group-level, dogs’ tolerance for delayed gratification was more within the range of tolerated delays found in rats^33^, crows^22^,^23^, ravens^23^,^30^ and capuchin monkeys^14^, thereby, showing that dogs possess only limited inhibitory control abilities in this food context. Hypotheses regarding the domestication of dogs (i.e. ‘emotional reactivity hypothesis^10^’ and ‘synergistic hypothesis^11^’) argue that dogs have been selected for an overall less reactive and consequently less impulsive personality type. Supporting this hypothesis many domesticated species excel their wild counterparts also in understanding human social cues (ferrets^34^, dogs (e.g. ref. 35), silver foxes^10^). Succeeding in such social tasks similarly requires individuals to inhibit searching for the food directly and instead first look at the human, implying that domesticated species might show enhanced inhibition skills compared to their wild counterparts^11^. Interestingly, our data only partially supports this hypothesis as only some dogs clearly showed enhanced inhibition in this delay of gratification paradigm, whereas, the majority of dogs was not able to tolerate high delays. However, in order to assess the effect of domestication on dogs’ inhibitory control it would be necessary to test also similarly raised wolves’, but this area has not received sufficient scientific attention so far and the only study that directly compared wolves’ and dogs’ performance in inhibitory control tasks found inconclusive results^38^. Another aspect to consider is that there was a large individual variation in dogs’ tolerance for delayed gratification in this test (i.e. ranging from 0 sec to 15 min). Such variation may be linked to other aspects of personality, as has been found in humans^36^ (e.g. temperament), hence futher research is also needed to investigate the interplay betweeen self-control and other personality traits.

Our second aim was to evaluate what aspects of the task and the dogs’ behaviour would affect their ‘waiting’ performance. Concerning the task, we found that the type of delayed reward had an effect on the dogs’ performance. Dogs tolerated higher delays when the reward increased in terms of quality, whereas it seemed to be more difficult for the dogs to wait if the reward increased in quantity. This finding is in line with other studies in cockatoos^21^, corvids^22^,^23^,^30^ and capuchin monkeys^31^, suggesting that animals in general are more sensitive to maximize rewards in terms of quality than quantity. Delaying a reward of higher quantity might simply require more inhibition than delaying a reward of higher quality since the initially offered option is more tempting to take if it is the same quality as the delayed option. This explanation is supported by our results, as dogs had fewer difficulties in waiting for the higher quantity of the less preferred reward than for the higher quantity of the more preferred reward type. This indicates that dogs acted more impulsively when the trial involved the highly preferred option as the immediate reward, whereas it facilitated waiting if the preferred reward was used as the delayed option.

Perhaps even more interesting is the fact that individual behavioural patterns during the delay task affected dogs’ capacity to wait for the preferred reward. Especially in the quality condition, in which subjects waited for longer, we found several behaviours associated with a better waiting performance in the test. Dogs that adopted a more distant position from the bowls, that laid down and that looked away from the food waited more often in the quality condition. Similarly, children that diverted their attention during the waiting period by for example, resting their heads on their arms or covering their eyes, were more successful in waiting than children that showed more ‘treat-focused’ behaviours, like holding, looking at or tasting the initial reward^24^,^25^,^37^. Such behaviours in the children literature have been referred to as ‘coping strategies^25^’, however, not only children show self-distracting behaviours to cope with impulsivity. Chimpanzees were shown to succeed more in a delay of gratification task if their enclosure was provided with toys than when no distractions were present, and crucially they engaged more in play behaviour when the food was within reach, than when it was visible but unobtainable^26^. Taken together, results of the study suggest chimpanzees were actively choosing to engage with the toys when they ‘most needed’ it and this helped them wait for the higher valued reward. Although we did not provide toys for the dogs in our study and did not directly test their capacity to ‘self-distract’, we did observe that dogs naturally coped differently with the imposed delay time. This is in line with observations from the previous study on dogs’ delay of gratification abilities^13^, however, in the previous study the dogs showed more active behaviour patterns (i.e. repetitive motor actions) compared to our current study. Also, a grey parrot displayed different diverting behaviours (e.g. grooming, looking away, closing eyes) while waiting for a better reward^27^, indicating that similar coping strategies might be found across animal taxa. Future studies on these aspects may be of particular interest.

In the quantity condition a similar pattern emerged but only looking away from the bowls facilitated waiting performance and only in the in higher delay stages. Interestingly, the opposite effect emerged in the shorter delays, with looking away hindering waiting performance. It is not altogether clear why this is the case, one possibility is that the task complexity was higher, requiring dogs to attend more to the food to work out the relative quantities, especially in the shorter delays at the beginning of the experiment. Another possibility is that such ‘coping strategies’ only emerge when the task becomes more complex i.e. longer delays are involved, or they emerge only once animals have had repeated experience with the task. Nevertheless it is clear that specific behaviours enacted by dogs allowed them to perform better.

Whether dogs exhibit these behaviours deliberately as a distraction tactic is debateable. The behaviours associated with the better waiting performance are also behaviours that could emerge as a by-product of remaining in the same position for a longer time. Dogs naturally lie down if they have to stay in the same area for a longer period. However, this would not explain why dogs moved away from the food again at the beginning of a new trial, as well as the fact that dogs were more successful if they looked away from the rewards. All dogs were still highly interested in the reward even after long delays, as shown in their immediate behavioural response (i.e. jumping up and running towards the bowl) when the more preferred reward was moved into the enclosure, which would rule out a decreased interest in the rewards after repeated sessions as an explanation for the observed behaviours (i.e. looking away and distant position).

Finally, our last aim was to investigate whether performance in the delay of gratification task would reflect the owner’s perception of their dogs’ self-control abilities in everyday life. To our surprise we did not find any correlation between the dogs’ impulsivity, as measured by a validated questionnaire^28^ and their performance in the delay of gratification task. This could indicate that the questionnaire and delay of gratification task do not measure the same underlying mechanism. Indeed, inhibitory control seems to be strongly context specific, as already shown by several studies (e.g. refs 1, 38 and 39). Individual performance in the delay of gratification paradigm may include an important motivational aspect in terms of the ‘values’ attributed by the individual to the different rewards, which may be an irrelevant aspect in a measure of self control based on dogs’ every day life experiences. This also supports the point of view that dogs’ inhibition abilities in daily life are possibly affected by learned behaviours based on reinforcement and punishment contingencies and/or human social inhibition and points to the importance of disentangling these aspects from dogs’ natural self-control abilities. This discrepancy between inhibition measurements also raises the question of whether there is any single valid measure of a species’ self-control. Future research needs to focus on validating test batteries composed of a number of tasks, which can be shown to measure inhibitory control consistently.

In conclusion the current study shows that at a group level dogs tolerated only moderate delays, however, some dogs showed enhanced waiting abilities. The dogs differentiated between qualitative and quantitative gains and renounced waiting early during a trial, suggesting their decision to wait took multiple factors into account. Moreover, some dogs exhibited behavioural patterns that predicted their waiting success for the delayed option, suggesting that like children, chimpanzees and parrots they may be able to use ‘self-distracting’ tactics.

## Methods

We tested 16 dogs (mean age + SD = 6.01 + 3.36 yrs.; see Table 2) of various breeds, including 9 mixed breed dogs in two different conditions (i.e. quality (low value vs. high value reward) and quantity (1 vs. 5 pieces of either low value or high value reward). Dogs were assigned to one of two groups, one starting with the quality condition (*N* = 8), the other with the quantity condition (*N* = 8; see Table 3 for an procedural overview). However, all dogs (except one) were tested in both conditions. The sex and age distribution was kept as similar as possible between the two groups. All procedures were discussed and approved by the ethics commission of the University of Veterinary Medicine Vienna in accordance with Good Scientific Practice guidelines and national legislation (Approval number: 10/12/97/2013). The study took place between April 2014 and March 2015 at the Clever Dog Lab, Messerli Research Institute, University of Veterinary Medicine of Vienna in Austria.

### Dog Impulsivity Assessment Scale

Prior to testing, dog owners were asked to fill in a questionnaire about their dog’s self-control ability in daily situations. The dog impulsivity assessment scale (DIAS) is a validated questionnaire^28^, consisting of 18 questions according to which the owner has to rank their dog’s behaviour on a 5-point scale. An overall self control score (=DIAS score) is obtained by summing up the rated answers and a higher score is thought to reflect higher impulsivity (see ref. 28 for details). Apart from this overall score, the questionnaire provides measures of three additional factors (i.e. behavioural regulation, aggressiveness and responsiveness). Since most dog owners were fluent in English (with the exception of one owner), we did not translate the questionnaire.

### Food Preference Test

Preference tests were run before testing to ensure that dogs clearly preferred one reward type and additionally, that all the dogs were able to differentiate between the reward quantities that were used. Owners were asked which food their dogs would eat but did not have a specific preference for (=low value reward (LVR)) and which rewards their dog preferred over everything else (=high value reward (HVR); see Table 2 for reward types used). To validate the owners’ suggestions, we first made sure that the dogs would eat the LVR and if they did we presented dogs with the two food types in a repeated two-choice test. The owner was instructed to sit on a chair and to keep the dog on a short leash in front of him/her. The experimenter kneeled in front of the dog (1.2 m distance) and visibly baited two plastic lids (10 cm diameter) with one piece of reward per lid. The lids were differently coloured (black and white) to facilitate reward discrimination. The black colour was always associated with the HVR and the white colour with the LVR. The experimenter lifted the baited lids and leaned forward to let the dog sniff the content of both lids while the owner restraint the dog from taking the rewards. After the dog had sniffed the content of both, the lids were placed on the ground (60–70 cm from dog and 50 cm distance between lids) and as soon as the experimenter moved her hands back to her lap, the dog was released. Only one choice was allowed and as soon as the dog had approached and eaten from one lid, the other was covered and moved back again. After the dog had eaten the reward, the owner called the dog back and the next trial started. 12 trials with alternating reward sides were conducted and in order to consider dogs choosing the HVR significantly more than chance level, we set criterion to 9 HVR-choices (one-tailed binomial: *p* < 0.02). If a dog did not reach criterion within 2 sessions other food types were chosen as rewards.

Before starting with the quantity condition (see Table 3 for an procedural overview), another preference test was conducted using the same methods as described above but this time with differences in reward quantity. Dogs had the choice between 1 piece of a reward (low quantity reward (LQR) on the white lid and 5 pieces (high quantity reward (HQR)) of the same reward quality on the black lid. Dogs have been shown to be able to differentiate between these quantities^40^. 12 trials were conduced for each reward type (first session only using LVR and second session only using HVR), alternating the side of the rewards in each trial. Criterion was again set to 9 HQR choices in each session and in case this criterion was not met within one session, a second session was conducted after a short break.

### Bowl Association

In the next step we familiarized the dog with the bowls used during the delay task. The previously established reward types were presented in two bowls (i.e. HVR in black and rectangular bowl and LVR in white and round bowl; both 10 cm diameter) attached to 1 m long sticks. Using these sticks allowed us, to move the rewards into the dog’s enclosure during testing, whilst the experimenter remained hidden behind a curtain. During the ‘bowl association’ trials, the dog was restraint by the owner while the experimenter visibly baited both bowls kneeling on the ground at a distance of 1.5 m from the dog (see Video 1). After the bowls were baited, they were moved towards the dog (within a 30 cm radius). The dog was allowed to sniff the content of both bowls while the owner restrained the dog from eating the rewards. Then, both bowls were simultaneously moved to positions equidistant to the dog (80 cm distance between bowls and 80 cm distance to dog). After the experimenter had removed her hands from the sticks, the dog was released and free to choose one bowl. As soon as the dog approached a bowl, the other was moved back and covered by the experimenter. The dog was allowed to eat the treat and then was called back by the owner and the next trial started. No choice criterion was set, since the purpose of these trials was simply to familiarize dogs with the bowls and their movements as well as build up the expectation of finding food within them. 12 trials were conducted; the side of rewards was counterbalanced and semi-randomized with the exception of having one reward no more than twice in a row on the same side. Two sessions were conducted, one before starting with the quality condition (LVR vs. HVR) and another before starting with the quantity condition (LQR vs. HQR). In the quantity session we conducted 6 trials with LVR rewards and 6 trials with HVR rewards, which were semi-randomly interspersed (no more than twice in a row).

### Apparatus

**Figure 2:**
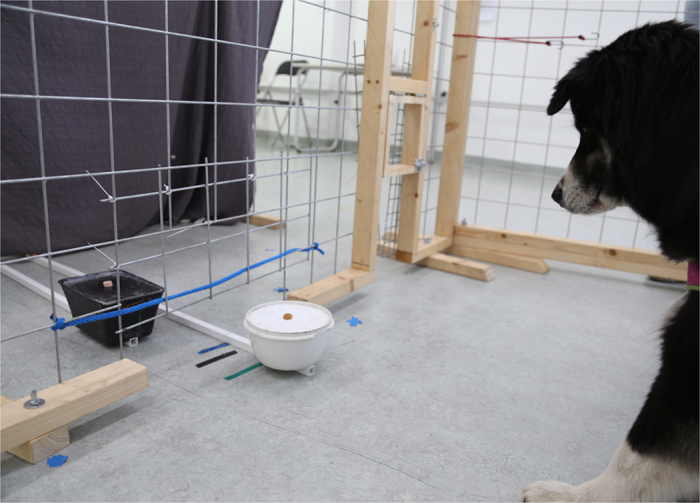
Test setup for quality condition. The dog is waiting for the black bowl, which contains the high value reward (in this case sausage) and resists from eating the low value reward (in this case dry food) from the white bowl.

The test enclosure consisted of three wooden fences with metal mesh positioned so as to form an area of approximately 4 m^2^ against the wall of one of the rooms in the Clever Dog Lab. The front fence was build so that a 20 cm space remained between the ground and the fence; this allowed us to manoeuvre the bowls in and out of the enclosure (see Fig. 2). A black curtain was positioned 40 cm from the enclosure behind which the experimenter was hidden during testing. The dog’s behaviour was observed via a webcam (connected to a laptop) mounted above the curtain.

### Training

Before each test condition we conducted training sessions, in order to familiarize dogs with the test procedure and the option of gaining the more preferred reward after a certain delay (see Video 2). During training sessions, the dog was kept on leash by the owner seated on a chair within the enclosure. Whilst sitting behind the curtain the experimenter baited both bowls and then pushed them simultaneously in front of the curtain (10 cm to front fence), so that the content was visible but not within reach of the dog. The training phase was conducted in the same way for the quality and quantity condition. The white bowl (containing always the less preferred reward (i.e. LVR or LQR)) was moved inside the enclosure first and following a 1 second delay the black bowl (containing always the more preferred reward (i.e. HVR or HQR) was also moved within the enclosure becoming available to the dog. Two types of trials were conducted during the training phase: *demonstration trials* and *training trials*. In *demonstration* trials, the owner was instructed to restrain the dog from eating out of the first bowl presented (LVR/LQR) and only release the dog (i.e. dropping the leash) when the second bowl (HVR/HQR) was moved within reach. During *training* trials, the dog was not restraint (i.e. loose leash) and the owner remained passive. In these training trials, if the dog took the LVR/LQR the second bowl was not made available. If the dog refrained from eating the LVR/LQR, the HVR/HQR was made available after the 1 s delay. The owner was instructed to always remain silent. Each training session consisted of 10 trials, following the same pattern: 3 demonstration trials, 3 training trials, 1 demonstration trial and 3 training trials. If a dog waited for the more preferred reward in at least 3 training trials, we considered it successful and the test condition with the first delay started. However, if this criterion was not reached another training session was conducted after a short break (Mean: quality: 1.53 sessions to criterion; quantity: 3 sessions to criterion). A maximum of 5 training sessions were conducted with no more than 3 sessions per test day. If a dog was not successful after these sessions, testing in this condition was terminated. This was the case for 3 dogs in the quantity condition and none in the quality condition.

### Testing

#### Demonstration Trials

Dogs that successfully passed the training went on to the test phase. Each test session started with 4 demonstration trials to accustom the dogs to the delay that they would experience during the subsequent test trials (see Video 2). Demonstration trials were conducted as during training. Once the demonstration trials were finished, the owner removed the leash and left the enclosure. The owners were hiding behind the curtain together with the experimenter throughout the test. One dog suffered from separation anxiety and thus the owner sat on the floor in front of the enclosure turning her back to the dog while remaining passive.

#### Test Trials

During the remaining 10 test trials, the dog had the free choice between the immediate less preferred reward (LVR in quality condition or LQR in quantity condition) or a delayed more preferred reward (HVR or HQR). In case of the quantity condition half of the trials consisted of a choice between 1 vs. 5 pieces of the LVR and the other half of trials were conducted with 1 vs. 5 pieces of the HVR. Those trials were randomly intermixed with each other with the exception of not having the same type of trials (i.e. LVR trial and HVR trial) more than twice in a row. Both baited bowls were always visible during trials and the delay started as soon as the white bowl entered the enclosure. If the dog chose the immediate reward, the other bowl was moved back immediately while the dog was allowed to eat the less preferred reward. However, if the dog refrained from eating the less preferred reward, the more preferred one was moved inside the enclosure after the delay. In this case the bowl containing the less preferred reward was withdrawn as soon as the dog started eating the more preferred reward. It was noted directly on a check-sheet whether the dog was successful during a trial. We tested dogs with the following delay stages: 2 s, 5 s, 10 s, 20 s, 30 s, 40 s, 50 s, 60 s, 70 s, 80 s, 110 s, 140 s, 170 s, 230 s, 460 s, 920 s and 1840 s. Dogs passed on to the next delay stage only if they waited for the more preferred bowl in at least 3 out of the 10 trials. If dogs did not reach this criterion, another session was conducted after a short break. Thus for each delay stage, dogs could experience a minimum of 1 test session (if they waited for the HVR/HQR on at least 3 of the 10 test trials) and a maximum of 4 test sessions (if they did not meet criterion). In the latter case, testing for that particular condition (quantity or quality) stopped and dogs either moved on to the subsequent condition, or were finished with the study. Depending on the dogs’ motivation one to three test sessions (Mean ± S.E.: 1.61 ± 0.67 test sessions) were conducted per day with a 3–5 min break between sessions in which the dogs were allowed to move freely in the test room.

### Data Analyses

All tests were recorded and the videos were analysed using Solomon coder beta (© 2015 by András Péter; http://solomoncoder.com/). The following behaviours were coded: choice (LVR/LQR = failure, HVR/HQR = success), latency to eat reward (i.e. the length of time from the moment the first bowl is pushed into the enclosure and the dog ate either the LVR or HVR), time spent in proximity to the available bowl during waiting time (close (within 20 cm to the bowl), medium (within 80 cm), distant (more than 80 cm)), the dog’s body posture during waiting time (i.e. durations of standing, sitting, laying) and the duration of looking away from the bowls (i.e. turning the head by approximately 90° away from the bowls after having looked at it first). Unfortunately, we could not analyse behaviours and latencies of 14% of all videos due to video malfunction; however, the success rate could be obtained from the data sheets. The questionnaires were analysed by hand and only the overall score (=DIAS) was used for subsequent analyses.

We analysed the data in R^41^ using the packages ‘lme4 (Version 1-1.7)^42^’ and ‘ordinal (Version 2015. 1-21)^43^’. A Cumulative link mixed model (CLMM) was utilized to analyse the influence of individual factors on the maximum delays reached. The model included the fixed factors: age (continuous), sex (factor), condition (quality, quantity), start condition (quality, quantity; factor) and DIAS score (continuous). Linear mixed models (LMM) were used to analyse the influence of behaviours during the waiting period on dogs’ success. The LMM were run with the success ratio as response variable (square root transformed). The success ratio was calculated by dividing the number of successful trials (=waiting) by the number of total trials (=10 trials, with the exception of two sessions, in which only 9 trials were conducted due to experimenter’s error). Remaining distant from the bowls, looking away and lying down were hypothesized as being behaviours that would positively affect waiting and were therefore included as explanatory factors. Therefore all LMMs were run with the fixed factors: delay stage (factor), ratio of trial time spent distant to the bowls, ratio of trial time spent in a laying position, ratio of trial time looking away from the bowls. To control for a possible depletion effect on dogs’ inhibition abilities^44^ we included the number of test sessions conducted per day (factor 1, 2, 3) as a factor in each model. We used the ratio of proximity to bowls as well as posture during the waiting time (i.e. duration of behaviour divided by total trial duration) since the trial length differed between sessions and dogs depending on the delay stage as well as on the decision to wait or not. In order investigate whether reward type influenced dogs’ success in the quantity condition we ran a separate model including the fixed effect of reward type (i.e. success ratio in LVR, success ration in HVR trials within each session). Models were selected using stepwise backward regression analyses to retain only significant effects in the model using likelihood ratio tests (LRT).

In order to investigate whether dogs give up waiting at a random point during the trial or whether they could anticipate the delay of the trial and consequently decide to give up waiting at the beginning of trials, we analysed dogs’ timing of errors (i.e. the time it took dogs to decide to give up waiting before the end of a trial). In order to do so, we used the same analysis as in previous studies (e.g. refs 13 and 29) and calculated the observed chance of giving up times using a Kaplan-Meier survival analysis. This analysis includes the giving up times of unsuccessful trials as well as the times of successful trials as censored data. Thus calculating the probability of each dog to wait longer than the already elapsed time within each trial. Against this observed distribution, we calculated the expected giving up times, which would be assumed under the null hypothesis to follow a constant chance of giving up during a trial. Then we compared the two distributions using an adjusted Kolmogorov-Smirnov test^45^.

20% of the videos were coded for reliability by a second independent coder (Inter-class correlation coefficient (ICC, consistency): proximity to bowls (close, medium, distant): all ICC < 0.71, looking away from bowls: ICC = 0.77, body posture during trials (sit, stand, lay): all ICC < 0.94)).

## Additional Information

**How to cite this article**: Brucks, D. *et al*. Reward type and behavioural patterns predict dogs’ success in a delay of gratification paradigm. *Sci. Rep.* **7**, 42459; doi: 10.1038/srep42459 (2017).

**Publisher's note:** Springer Nature remains neutral with regard to jurisdictional claims in published maps and institutional affiliations.

## Supplementary Material

Supplementary Material

Supplementary Video 1

Supplementary Video 2

## Figures and Tables

**Table 1:** Effects of delay, looking away, lying and distant position on dogs’ waiting success comparing the quality and the quantity condition.

Factors	Quality Condition	Quantity Condition
Delay	*χ*^*2*^ (12) = 118.13, ***p*** **< 0.001**	Interaction: *χ*^*2*^ (10) = 37.28, ***p*** **< 0.001**
Look away	*χ*^*2*^ (1) = 9.27, ***p*** **= 0.002**	
Lying	*χ*^*2*^ (1) = 48.11, ***p*** **< 0.001**	*χ*^*2*^ (1) = 0.77, *p* = 0.380
Distant	*χ*^*2*^ (1) = 7.66, ***p*** **= 0.006**	*χ*^*2*^ (1) = 0.06, *p* = 0.802
Tests per day	*χ*^*2*^ (2) = 2.92, *p* = 0.231	*χ*^*2*^ (2) = 2.47, *p* = 0.291

Results from LMMs are depicted (see Tables S2 and S3 for model estimates).

Significant results are given in bold.

**Table 2:** Individual characteristics and DIAS-Score of dogs that participated in the study (*N* = 16).

Name	Sex	Age (years)	Breed	LVR	HVR	1^st^ Condition	DIAS Score^1^
Akina	F	5.8	Akita Inu	Dry food	Sausage	Quantity	0.565
Buck	M	4.9	Beagle	Apple	Dry food	Quantity	0.500
Cameron*	M	1.9	Border Collie	Dry food	Sausage	Quantity	0.478
Gizmo	M	8.1	Chihuahua-Mix	Dry food	Sausage	Quantity	0.633
Hybie	F	5.3	Labrador-Mix	Dry food	Sausage	Quantity	0.644
Kilio^x^	M	4.3	Terrier-Mix	Dry food	Sausage	Quality	0.553
Lola	F	1.3	Border Collie-Mix	Cucumber	Sausage	Quantity	0.552
Luna	F	1.5	Siberian Husky	Dry food	Sausage	Quality	0.663
Melissa	F	12.2	Shepherd-Mix	Bread	Sausage	Quality	0.506
Michel	M	8.9	Mixed breed	Carrot	Sausage	Quality	0.511
Nash^x^	M	9.9	German Shepherd	Dry food	Sausage	Quantity	0.511
Sokrates	M	8.1	Bardino-Mix	Dry food	Sausage	Quality	0.378
Talie	M	3.0	Siberian Husky	Dry food	Sausage	Quality	0.544
Teddy	M	7.0	Shepherd-Mix	Dry food	Cheese	Quality	0.667
Todor^x^	M	10.8	Mixed breed	Dry food	Sausage	Quantity	0.389
Ultimo	M	4.5	Border Collie	Dry food	Sausage	Quality	0.500

^*^Only tested in quantity condition.

^x^Dogs did not pass training criterion in quantity condition.

^1^The higher the score the higher the assumed impulsivity.

LVR = low value reward.

HVR = high value reward.

**Table 3:** Overview of testing procedure.

**Group 1** (*N* = 8)	Food preference test	Bowl association quality	*Quality condition*	Quantity preference test	Bowl association quantity	*Quantity condition*
**Group 2** (*N* = 8)*	Food preference test	Quantity preference test	Bowl association quantity	***Quantity condition***	Bowl association quality	***Quality condition***

^*^One dog within this group was only tested in the quantity condition.
