# Supplementary material for: Reward type and behavioural patterns predict dogs’ success in a delay of gratification paradigm
Source: Sci Rep. 2017 Mar 8;7:42459. doi: 10.1038/srep42459 (PMC5341119; doi:10.1038/srep42459)
Supplement: Supplementary Material [file srep42459-s1.docx]

**Reward type and behavioural patterns predict dogs’ success in a delay of gratification paradigm**

**Désirée Brucks^1^*, Matteo Soliani^1^, Friederike Range^1^, Sarah Marshall-Pescini^1^**

^1^ Comparative Cognition, Messerli Research Institute, University of Veterinary Medicine Vienna, Medical University of Vienna, University of Vienna, 1210 Vienna, Austria

* desiree.brucks@vetmeduni.ac.at

**Supplementary Material**

**Video 1.** This video shows the food preference test and bowl-association phase.

**Video 2.** This video shows the test procedure (i.e. demonstration trials and test trials).

**Table S1.** Individual DIAS-Scores of dogs that participated in the study (*N* = 16)

| Name | Sex | Age (years) | Breed | DIAS Score^1^ | Behavioural regulation | Aggressiveness | Responsiveness |
| --- | --- | --- | --- | --- | --- | --- | --- |
| Akina | F | 5.8 | Akita Inu | 0.565 | 0.54 | 0.56 | 0.45 |
| Buck | M | 4.9 | Beagle | 0.500 | 0.54 | 0.36 | 0.68 |
| Cameron | M | 1.9 | Border Collie | 0.478 | 0.40 | 0.52 | 0.84 |
| Gizmo | M | 8.1 | Chihuahua-Mix | 0.633 | 0.68 | 0.32 | 0.52 |
| Hybie | F | 5.3 | Labrador-Mix | 0.644 | 0.60 | 0.36 | 0.88 |
| Kilio^x^ | M | 4.3 | Terrier-Mix | 0.553 | 0.58 | 0.50 | 0.56 |
| Lola | F | 1.3 | Border Collie-Mix | 0.552 | 0.52 | 0.40 | 0.72 |
| Luna | F | 1.5 | Siberian Husky | 0.663 | 0.64 | 0.36 | 0.84 |
| Melissa | F | 12.2 | Shepherd-Mix | 0.506 | 0.50 | 0.48 | 0.45 |
| Michel | M | 8.9 | Mixed breed | 0.511 | 0.42 | 0.52 | 0.64 |
| Nash | M | 9.9 | German Shepherd | 0.511 | 0.52 | 0.20 | 0.80 |
| Sokrates | M | 8.1 | Bardino-Mix | 0.378 | 0.26 | 0.44 | 0.52 |
| Talie | M | 3.0 | Siberian Husky | 0.544 | 0.46 | 0.52 | 0.68 |
| Teddy | M | 7.0 | Shepherd-Mix | 0.667 | 0.62 | 0.68 | 0.64 |
| Todor | M | 10.8 | Mixed breed | 0.389 | 0.30 | 0.44 | 0.56 |
| Ultimo | M | 4.5 | Border Collie | 0.500 | 0.42 | 0.36 | 1.00 |

^1^ The higher the score the higher the assumed impulsivity.

**Table S2.** Effects of delay, looking away, laying and distant position on dogs’ success in the quality condition. Estimates and test statistics are based on REML t-tests. Success probability was modelled using a LMM (Satterthwaite approximation) with subject as random factor.

| **Fixed Effects** | **Estimate** | **S.E.** | **β** | ***t-value*** | ***P*** |
| --- | --- | --- | --- | --- | --- |
| Intercept | 0.542 | 0.112 | 16.41 | 4.836 | < 0.001 |
| Delay 5s | -0.158 | 0.067 | 111.85 | -2.380 | 0.019 |
| Delay 10s | -0.264 | 0.075 | 113.46 | -3.530 | < 0.001 |
| Delay 20s | -0.489 | 0.081 | 112.69 | -6.010 | < 0.001 |
| Delay 30s | -0.657 | 0.081 | 113.24 | -8.151 | < 0.001 |
| Delay 40s | -0.659 | 0.097 | 111.46 | -6.777 | < 0.001 |
| Delay 50s | -0.627 | 0.094 | 112.09 | -6.669 | < 0.001 |
| Delay 60s | -0.875 | 0.104 | 111.44 | -8.388 | < 0.001 |
| Delay 70s | -0.739 | 0.097 | 111.69 | -7.588 | < 0.001 |
| Delay 80s | -0.774 | 0.100 | 111.52 | -7.751 | < 0.001 |
| Delay 110s | -1.051 | 0.137 | 109.99 | -7.667 | < 0.001 |
| Delay 140s | -1.219 | 0.119 | 110.56 | -10.267 | < 0.001 |
| Delay 170s | -1.234 | 0.124 | 111.22 | -9.936 | < 0.001 |
| Ratio look away | 0.599 | 0.206 | 109.12 | 2.910 | 0.004 |
| Ratio laying | 0.491 | 0.069 | 110.31 | 7.145 | < 0.001 |
| Ratio distant | 0.636 | 0.241 | 108.83 | 2.638 | 0.010 |

**Table S3.** Effects of delay and looking away behaviour on dogs’ success in the quantity condition. Estimates and test statistics are based on REML t-tests. Success probability was modelled using a LMM (Satterthwaite approximation) with subject as random factor.

| **Fixed Effects** | **Estimate** | **S.E.** | **β** | ***t-value*** | ***P*** |
| --- | --- | --- | --- | --- | --- |
| Intercept | 0.344 | 0.117 | 11.85 | 2.936 | 0.013 |
| Delay 5s | -0.069 | 0.081 | 46.48 | -0.854 | 0.397 |
| Delay 10s | -0.231 | 0.079 | 44.62 | -2.912 | 0.006 |
| Delay 20s | -0.296 | 0.084 | 46.86 | -3.540 | < 0.001 |
| Delay 30s | -0.224 | 0.127 | 44.80 | -1.770 | 0.084 |
| Delay 40s | 0.043 | 0.114 | 45.42 | 0.378 | 0.707 |
| Delay 50s | -0.152 | 0.192 | 44.11 | -0.794 | 0.432 |
| Delay 60s | -0.264 | 0.169 | 44.38 | -1.565 | 0.125 |
| Delay 70s | -0.519 | 0.157 | 43.54 | -3.314 | 0.002 |
| Delay 80s | -0.304 | 0.180 | 44.17 | -1.692 | 0.098 |
| Delay 110s | -0.140 | 0.182 | 44.15 | -0.773 | 0.444 |
| Delay 140s | -0.853 | 0.105 | 46.59 | -8.147 | < 0.001 |
| Delay 170s | -0.993 | 0.113 | 44.72 | -8.763 | < 0.001 |
| Ratio look away | 0.469 | 0.251 | 42.32 | 1.934 | 0.060 |
| Delay 10s*ratio look away | 2.354 | 1.171 | 42.55 | 2.011 | 0.051 |
| Delay 20s*ratio look away | -0.087 | 0.391 | 42.65 | -0.223 | 0.825 |
| Delay 30s*ratio look away | -0.720 | 1.266 | 42.56 | -0.569 | 0.573 |
| Delay 40s*ratio look away | -2.428 | 0.639 | 42.48 | -3.800 | < 0.001 |
| Delay 50s*ratio look away | -1.049 | 2.308 | 42.66 | -0.454 | 0.652 |
| Delay 60s*ratio look away | 0.607 | 3.449 | 42.65 | 0.176 | 0.862 |
| Delay 70s*ratio look away | 4.439 | 2.021 | 42.64 | 2.197 | 0.034 |
| Delay 80s*ratio look away | 0.365 | 0.978 | 42.65 | 0.373 | 0.711 |
| Delay 110s*ratio look away | -0.861 | 0.648 | 42.62 | -1.330 | 0.191 |
| Delay 140s*ratio look away | 1.426 | 0.787 | 42.54 | 1.812 | 0.077 |

**Table S4.** Effects of delay and reward type on dogs’ success in the quantity condition. Estimates and test statistics are based on REML t-tests. Success probability was modelled using a LMM (Satterthwaite approximation) with subject as random factor.

| **Fixed Effects** | **Estimate** | **S.E.** | **β** | ***t-value*** | ***P*** |
| --- | --- | --- | --- | --- | --- |
| Intercept | 0.542 | 0.112 | 16.41 | 4.836 | < 0.001 |
| Delay 5s | -0.158 | 0.067 | 111.85 | -2.380 | 0.019 |
| Delay 10s | -0.264 | 0.075 | 113.46 | -3.530 | < 0.001 |
| Delay 20s | -0.489 | 0.081 | 112.69 | -6.010 | < 0.001 |
| Delay 30s | -0.657 | 0.081 | 113.24 | -8.151 | < 0.001 |
| Delay 40s | -0.659 | 0.097 | 111.46 | -6.777 | < 0.001 |
| Delay 50s | -0.627 | 0.094 | 112.09 | -6.669 | < 0.001 |
| Delay 60s | -0.875 | 0.104 | 111.44 | -8.388 | < 0.001 |
| Delay 70s | -0.739 | 0.097 | 111.69 | -7.588 | < 0.001 |
| Delay 80s | -0.774 | 0.100 | 111.52 | -7.751 | < 0.001 |
| Delay 110s | -1.051 | 0.137 | 109.99 | -7.667 | < 0.001 |
| Delay 140s | -1.219 | 0.119 | 110.56 | -10.267 | < 0.001 |
| Delay 170s | -1.234 | 0.124 | 111.22 | -9.936 | < 0.001 |
| Type (LVR) | 0.599 | 0.206 | 109.12 | 2.910 | 0.004 |

**Table S5.** Maximum values from Kolmogorov-Smirnoff Test when comparing expected and observed distribution of giving up times in both conditions showing error times in each session. If difference is significant it indicates that the dogs renounced waiting earlier than expected by a constant giving up chance.

|  |  |  | **Delay stage (seconds)** | | | | | | | | | | | | | | |
| --- | --- | --- | --- | --- | --- | --- | --- | --- | --- | --- | --- | --- | --- | --- | --- | --- | --- |
| **Dog** | **Cond** | **S** | **10** | **20** | **30** | **40** | **50** | **60** | **70** | **80** | **110** | **140** | **170** | **230** | **460** | **920** | **1840** |
| Akina | QUAL | 1 | NS | - | - | - | - | - | - | - | - | - | - | - | - | - | - |
|  |  | 2 | NS | - | - | - | - | - | - | - | - | - | - | - | - | - | - |
|  |  | 3 | NS | - | - | - | - | - | - | - | - | - | - | - | - | - | - |
|  |  | 4 | NS | - | - | - | - | - | - | - | - | - | - | - | - | - | - |
|  | QUAN | 1 | **1.23** | - | - | - | - | - | - | - | - | - | - | - | - | - | - |
|  |  | 2 | NS | - | - | - | - | - | - | - | - | - | - | - | - | - | - |
|  |  | 3 | NS | - | - | - | - | - | - | - | - | - | - | - | - | - | - |
|  |  | 4 | NS | - | - | - | - | - | - | - | - | - | - | - | - | - | - |
| Buck | QUAL | 1 | **1.03** | 0.56 | **1.08** | - | - | - | - | - | - | - | - | - | - | - | - |
|  |  | 2 | - | - | NS | - | - | - | - | - | - | - | - | - | - | - | - |
|  |  | 3 | - | - | NS | - | - | - | - | - | - | - | - | - | - | - | - |
|  |  | 4 | - | - | NS | - | - | - | - | - | - | - | - | - | - | - | - |
| Cameron | QUAN | 1 | 0.79 | **1.32** | NA | 0.47 | 0.60 | 0.31 | **1.83** | S | 0.31 | S | S | **2.21** | **1.11** | **1.00** | NS |
|  |  | 2 | 0.56 | - | - | - | - | - | - | - | - | - | - | - | S | - | NS |
|  |  | 3 | - | - | - | - | - | - | - | - | - | - | - | - | - | - | - |
|  |  | 4 | - | - | - | - | - | - | - | - | - | - | - | - | - | - | - |
| Gizmo | QUAL | 1 | S | 0.05 | 0.76 | **1.60** | S | 0.94 | 0.31 | 0.62 | **1.84** | 1.02 | NS | - | - | - | - |
|  |  | 2 | - | - | - | 0.58 | - | - | - | - | - | - | NS | - | - | - | - |
|  |  | 3 | - | - | - | - | - | - | - | - | - | - | NS | - | - | - | - |
|  |  | 4 | - | - | - | - | - | - | - | - | - | - | NS | - | - | - | - |
|  | QUAN | 1 | 0.63 | **1.62** | - | - | - | - | - | - | - | - | - | - | - | - | - |
|  |  | 2 | NS | NS | - | - | - | - | - | - | - | - | - | - | - | - | - |
|  |  | 3 | - | NS | - | - | - | - | - | - | - | - | - | - | - | - | - |
|  |  | 4 | - | NS | - | - | - | - | - | - | - | - | - | - | - | - | - |
| Kilio | QUAL | 1 | 0.64 | - | - | - | - | - | - | - | - | - | - | - | - | - | - |
|  |  | 2 | NS | - | - | - | - | - | - | - | - | - | - | - | - | - | - |
|  |  | 3 | NS | - | - | - | - | - | - | - | - | - | - | - | - | - | - |
|  |  | 4 | 0.64 | - | - | - | - | - | - | - | - | - | - | - | - | - | - |
| Luna | QUAL | 1 | 0.42 | S | 0.56 | 0.31 | 0.62 | **1.03** | **2.05** | **1.73** | NS | NA | - | - | - | - | - |
|  |  | 2 | - | - | - | - | - | NS | **1.13** | NA | **1.76** | NA | - | - | - | - | - |
|  |  | 3 | - | - | - | - | - | - | - | - | - | NS | - | - | - | - | - |
|  |  | 4 | - | - | - | - | - | - | - | - | - | NS | - | - | - | - | - |
|  | QUAN | 1 | 0.08 | S | 0.64 | **1.62** | **1.24** | 0.92 | **2.13** | 0.53 | 0.87 | **1.69** | NS | - | - | - | - |
|  |  | 2 | - | - | - | 0.30 | - | - | 0.39 | - | - | - | 0.69 | - | - | - | - |
|  |  | 3 | - | - | - | - | - | - | - | - | - | - | NS | - | - | - | - |
|  |  | 4 | - | - | - | - | - | - | - | - | - | - | NS | - | - | - | - |
| Michel | QUAL | 1 | S | S | S | S | 0.51 | S | 0.63 | 0.31 | NA | NA | - | - | - | - | - |
|  |  | 2 | - | - | - | - | - | - | - | - | - | NS | - | - | - | - | - |
|  |  | 3 | - | - | - | - | - | - | - | - | - | NS | - | - | - | - | - |
|  |  | 4 | - | - | - | - | - | - | - | - | - | NS | - | - | - | - | - |
|  | QUAN | 1 | NA | NS | - | - | - | - | - | - | - | - | - | - | - | - | - |
|  |  | 2 | - | NS | - | - | - | - | - | - | - | - | - | - | - | - | - |
|  |  | 3 | - | NS | - | - | - | - | - | - | - | - | - | - | - | - | - |
|  |  | 4 | - | NS | - | - | - | - | - | - | - | - | - | - | - | - | - |
| Sokrates | QUAL | 1 | **1.43** | NS | - | - | - | - | - | - | - | - | - | - | - | - | - |
|  |  | 2 | - | NS | - | - | - | - | - | - | - | - | - | - | - | - | - |
|  |  | 3 | - | NS | - | - | - | - | - | - | - | - | - | - | - | - | - |
|  |  | 4 | - | NS | - | - | - | - | - | - | - | - | - | - | - | - | - |

| Talie | QUAL | 1 | S | S | S | **1.46** | **2.45** | - | - | - | - | - | - | - | - | - | - |
| --- | --- | --- | --- | --- | --- | --- | --- | --- | --- | --- | --- | --- | --- | --- | --- | --- | --- |
|  |  | 2 | - | - | - | - | NS | - | - | - | - | - | - | - | - | - | - |
|  |  | 3 | - | - | - | - | NS | - | - | - | - | - | - | - | - | - | - |
|  |  | 4 | - | - | - | - | NS | - | - | - | - | - | - | - | - | - | - |
|  | QUAN | 1 | NA | **1.22** | 0.61 | S | 0.81 | **1.78** | 0.30 | **1.50** | **1.07** | NS | - | - | - | - | - |
|  |  | 2 | - | - | - | - | - | - | - | - | - | NS | - | - | - | - | - |
|  |  | 3 | - | - | - | - | - | - | - | - | - | NS | - | - | - | - | - |
|  |  | 4 | - | - | - | - | - | - | - | - | - | NA | - | - | - | - | - |
| Teddy | QUAL | 1 | **1.08** | NS | NS | NS | **1.08** | NS | NS | NS | - | - | - | - | - | - | - |
|  |  | 2 | - | NS | NS | NS | - | NS | NS | NS | - | - | - | - | - | - | - |
|  |  | 3 | - | **1.68** | 0.88 | NA | - | NS | NS | NS | - | - | - | - | - | - | - |
|  |  | 4 | - | - | NA | - | - | **1.43** | **1.22** | NS | - | - | - | - | - | - | - |
|  | QUAN | 1 | 0.90 | **1.72** | **1.41** | 0.47 | **1.70** | 0.82 | NA | NA | NA | NA | - | - | - | - | - |
|  |  | 2 | - | - | - | - | - | - | - | NA | - | NS | - | - | - | - | - |
|  |  | 3 | - | - | - | - | - | - | - | - | - | NS | - | - | - | - | - |
|  |  | 4 | - | - | - | - | - | - | - | - | - | NS | - | - | - | - | - |
| Todor | QUAL | 1 | 0.51 | **1.56** | **2.01** | - | - | - | - | - | - | - | - | - | - | - | - |
|  |  | 2 | - | **1.28** | **1.86** | - | - | - | - | - | - | - | - | - | - | - | - |
|  |  | 3 | - | **1.83** | NS | - | - | - | - | - | - | - | - | - | - | - | - |
|  |  | 4 | - | - | **1.53** | - | - | - | - | - | - | - | - | - | - | - | - |

NA = video is missing

- = not tested

S = 100% success

NS = not successful (i.e. no waiting in any trial, consequently dogs did not experience delay duration and could not be analysed)

Critical values written in bold = 1.03 for *P* < 0.05; 1.21 for *P* < 0.01; 1.42 for *P* < 0.001 (from Haccou & Meelis 1992)
